# Key Neuropeptides Regulating Molting in Pacific White Shrimp (*Penaeus vannamei*): Insights from Transcriptomic Analysis

**DOI:** 10.3390/ani15040540

**Published:** 2025-02-13

**Authors:** Xianliang Li, Yunjiao Li, Zecheng Li, Hu Chen

**Affiliations:** 1 Key Laboratory of Tropical Hydrobiology and Biotechnology of Hainan Province, Hainan Aquaculture Breeding Engineering Research Center, School of Marine Biology and Fisheries, School of Breeding and Multiplication (Sanya Institute of Breeding and Multiplication), Hainan University, Haikou 570228, China; 20192111310021@hainanu.edu.cn (X.L.); lizecheng@hainanu.edu.cn (Z.L.); 2 Fisheries Research Institute of Sichuan Academy of Agricultural Sciences, Yibin 644000, China; yunjiaoli@scsaas.cn

**Keywords:** *Penaeus vannamei*, molting, neuropeptides, transcriptomic neuropeptides, transcriptomic

## Abstract

Molting is a vital physiological process essential for the growth and development of *Penaeus vannamei*, with significant implications for aquaculture productivity. This study aimed to identify neuropeptide-related genes involved in molting through transcriptomic analysis. RNA sequencing of pre-molt and post-molt samples revealed 1203 differentially expressed genes (DEGs). Functional enrichment analysis indicated that these genes play significant roles in cuticle formation and molting regulation. Among the DEGs, 243 were predicted to be neuropeptides based on the presence of signal peptides and the absence of transmembrane domains. Five key neuropeptide genes—*PvCHH*, *PvMIH*, *PvEH* I, *PvCDA* I, and *PvCDA* II—were identified as critical regulators of molting. Their role was further validated through RT-qPCR analysis, confirming their close association with the molting process. These genes were highlighted in this study as pivotal factors driving molting in *P. vannamei.* The neuropeptides identified in this research are anticipated to offer valuable insights into the regulation of molting. Additionally, their synthetic products hold promise for improving molting consistency in shrimp aquaculture.

## Simple Summary

This study explored the involvement of neuropeptide-related genes in the molting process of *Penaeus vannamei* through comprehensive transcriptomic analysis. High-quality transcriptomic data were generated using RNA sequencing, revealing 1203 differentially expressed genes (DEGs) between the pre-molt and post-molt stages. Functional enrichment analysis demonstrated that these DEGs were primarily associated with molecular functions such as binding, catalytic activity, and structural constituents of the cuticle. Emphasis was placed on neuropeptides, leading to the identification of 243 differentially expressed neuropeptides. These neuropeptides, characterized by the presence of signal peptides and the absence of transmembrane domains, were found to play critical roles in the regulation of molting. Furthermore, five key neuropeptide genes—*PvCHH*, *PvMIH*, *PvEH I*, *PvCDA I*, and *PvCDA II*—were identified, including novel sequences that had hitherto not been annotated in *P. vannamei*. The expression patterns of these genes were validated using RT-qPCR, with results consistent with the transcriptomic data. This study highlights the significance of neuropeptide-related pathways in the regulation of molting and establishes a foundation for further functional studies. Our findings enhance our understanding of the molecular mechanisms governing molting in *P. vannamei* and provide valuable insights that could inform future research in shrimp physiology and aquaculture practices.

## 1. Introduction

The white shrimp (*Penaeus vannamei*) is the most economically significant crustacean globally, both in terms of farming scale and production. With a market value nearing US$30 billion, the industry continues to experience rapid and sustained growth. According to the Food and Agriculture Organization of the United Nations (FAO), global white shrimp production in 2020 exceeded 5.81 million tons, accounting for about 80% of all farmed crustaceans [1,2]. The transformation of white shrimp aquaculture from traditional small-scale operations to a global industry has been driven by advancements in breeding programs, nutritional research, and innovations in farming technologies. These developments have laid a strong foundation for the industry’s continued high-quality growth [3,4,5]. Building on this progress and considering the average annual growth rate of around 5% [2], it is projected that annual white shrimp production will reach 10 million tons within the next decade. Given the scale of this industry, refined physiological regulation in aquaculture has become a critical requirement for advancing farming technologies. However, there remains a significant gap in research on various fundamental aspects of shrimp physiology, such as the regulation of molting [6,7].

The molting physiology of white shrimp plays a critical role in their production, growth, and reproduction. Throughout their lifetime, white shrimp undergo approximately 50 molts [8]. Their development begins with metamorphosis from eggs to mysid larvae, a process that involves 11–12 molts. Following this, the larvae enter a growth phase, during which they molt repeatedly to mature. This stage, known as growth molting, occurs 12 times more frequently in developmental stages compared to adult growth molts [9]. In addition, broodstock must undergo reproductive molting prior to mating. The molting cycle varies depending on the size of the shrimp. For instance, 2 g white shrimp typically experience a molting cycle of about 5 days, while 15 g shrimp have a longer cycle of approximately 11 days [10]. In crustaceans, the molting cycle is generally divided into four main phases: inter-molt (C), pre-molt (D), ecdysis (E), and post-molt (A/B). However, in white shrimp, the process can be further categorized into eight distinct stages based on morphological changes in the development of the caudal limbs. These stages include inter-molt (C), onset of pre-molt (D0), early pre-molt (D1), intermediate pre-molt (D2), late pre-molt (D3), ecdysis (E), early post-molt (A), and late post-molt (B). For example, the pre-molt phase (D0–D3) involves preparations for shedding the old exoskeleton, while ecdysis, the actual shedding of the cuticle, is a rapid process that lasts only seconds to minutes. The post-molt stages (A/B) are critical for the hardening of the new exoskeleton, which provides essential protection and structural support [6]. Given the significance of molting across various stages of shrimp production and its intricate nature, a deeper understanding of its regulatory mechanisms could greatly enhance the efficiency and sustainability of the white shrimp farming industry.

In shrimp, the regulation of molting is primarily governed by a neuroendocrine system that integrates neuropeptide signals, ensuring the proper timing and execution of the molting cycle [11]. Central to this process are the eyestalk neurosecretory complexes, which release molt-inhibiting hormone (MIH) [7,12]. This neuropeptide plays a crucial role in delaying molting by inhibiting the release of ecdysteroids, which are molting hormones produced by the Y-organs [12]. When MIH levels decrease, the Y-organs secrete ecdysteroids, initiating the pre-molt phase. Other key neuropeptides involved in this process include crustacean hyperglycemic hormone (CHH) and mandibular organ-inhibiting hormone (MOIH), both of which modulate energy metabolism and molting progression [13]. Research has highlighted the role of neuropeptides such as bursicon, which contributes to cuticle hardening post-ecdysis in shrimp [14]. Despite these advances, the regulation of shrimp molting remains highly complex, and growing evidence suggests that additional key molting factors have yet to be identified.

Effective molting management in shrimp farming is crucial for improving survival rates and overall productivity. Research has demonstrated that inadequate molting management can result in significant losses, particularly when molting occurs asynchronously, leaving shrimp vulnerable to attacks and cannibalism by their counterparts [15]. Encouraging synchronized molting mitigates these risks and improves farm efficiency. Neuropeptides play a key role in regulating shrimp molting [7], presenting substantial commercial potential as targets for synchronization strategies. For instance, they could be harnessed to monitor molting progress and identify influencing factors. Furthermore, the use of synthetic analogs may help standardize the molting process, thereby improving the efficiency of shrimp aquaculture practices. However, research on the role of neuropeptides in regulating molting in white shrimp remains limited. Recent advances in transcriptomics and proteomics have shed light on the molecular pathways and regulatory networks involved in this process [16,17], offering a promising avenue for further exploration of neuropeptide-mediated molting control. By leveraging transcriptomics, researchers can identify key genes and neuropeptides involved in molting regulation, providing a molecular foundation for developing effective molting management techniques. Such advancements have the potential to reduce economic losses in shrimp farming and contribute to more sustainable and efficient aquaculture practices.

In this study, we sought to identify key neuroregulatory peptides involved in molting regulation through transcriptomic analysis, with emphasis on their relevance to aquaculture practices. Given the critical role of molting management in shrimp farming, our research specifically targeted the pre-molt period (D stage), particularly the D2–D3 sub-stages, which are most closely associated with successful molting outcomes. Neuropeptide levels during these stages are of paramount importance, as evidenced by our previous research on eclosion hormones (EH) [18]. Therefore, shrimp in the pre-molt (D2–D3) and post-molt (A–B) stages were selected as the experimental and control groups, respectively. A reference transcriptome was then employed to identify all differentially expressed genes (DEGs) before and after molting. These DEGs were annotated using Gene Ontology (GO) and Kyoto Encyclopedia of Genes and Genomes (KEGG) pathway analyses. Further screening was conducted to identify potential neuropeptides based on structural characteristics, such as transmembrane domains and signal peptides. From the GO dataset, we selected the most critical neuropeptides involved in multiple pathways. The expression changes in these neuropeptides during the pre- and post-molt stages were subsequently validated using RT-qPCR. The findings of this study are expected to identify key neuropeptides that potentially regulate molting, which could serve as targets for synchronized molting control in Pacific white shrimp aquaculture, contributing to the optimization of shrimp farming strategies.

## 2. Materials and Methods

### 2.1. Aquaculture and Sampling Procedures

The three-month-old *P. vannamei* (with a mean body length of 6.5 ± 1 cm and body weight of 2.6 ± 1 g) in the pre-molt (D2–D3) and post-molt (A–B) stages were carefully selected for this study. The D2–D3 stages (pre-molt) were chosen as the experimental group since they represent a critical phase in the molting cycle, during which molting-related physiological processes are most active. At this stage, key hormones, such as ecdysteroids reach their peak levels, driving essential processes like cuticle separation, new cuticle synthesis, and tissue remodeling. This made the D2–D3 stages an ideal period to study the molecular and hormonal mechanisms that govern the molting process. In contrast, the A–B stages (post-molt) were selected as the control group because they represent the recovery phase following molting. During this phase, hormonal activity subsides, and the focus shifts to cuticle hardening and calcification. The relatively stable conditions of the A–B stages provide a baseline for comparison with the dynamic changes observed in the D2–D3 stages. This comparison is crucial for identifying regulatory factors specific to the molting process.

The shrimp assigned to the designated groups were anesthetized on ice and promptly dissected to extract the nervous system, which comprised the eye stalk, brain ganglion, peritrophic nerve ganglion, thoracic nerve chain, and abdominal nerve chain. Nerve tissues from five shrimp were pooled to form a single sample, and two such samples were collected from each experimental group. These samples were initially preserved in an animal tissue preservation solution overnight and subsequently stored at −80 °C. Throughout the experiment, the culture water was maintained at a temperature of 26 °C, with a salinity of 28 and a pH of 7.9 ± 0.5. To ensure water quality, water was changed once daily, with two-thirds of the total volume replaced each time.

### 2.2. RNA Extraction and Transcriptome Sequencing

The total RNA from the samples was extracted using the Trizol method. The concentration, purity, and integrity of the RNA were assessed through agarose gel electrophoresis and the Agilent 2100 Bioanalyzer (Agilent, Santa Clara, CA, USA). To ensure the quality of the RNA, the following criteria were met: the total RNA amount was ≥1 μg, the concentration was ≥35 ng/μL, the OD260/280 ratio was between 1.8 and 2.2, the OD260/230 ratio was ≥2.0, and the RNA Integrity Number (RIN) was >6.5. Subsequently, mRNA was isolated by capturing the poly-A tails of mRNA using oligo(dT) magnetic beads. The mRNA was then fragmented into smaller pieces by adding a fragmentation buffer. Fragments ranging from 250 to 300 bp were selected using magnetic beads. First-strand cDNA was synthesized using random hexamers and reverse transcriptase, followed by the synthesis of the second strand. The cDNA ends were repaired using an end repair mix to create blunt ends, and an “A” base was added to the 3′ ends to facilitate ligation with Y-shaped adapters. Finally, the libraries were prepared in accordance with the sequencing requirements provided by Shanghai Meiji Biopharmaceutical Technology Co., Ltd., (Shanghai, China) and transcriptome sequencing was performed.

For the raw sequencing data (Accession: PRJNA1195882), quality control was performed using the software fastp v0.23.4, (available at https://github.com/OpenGene/fastp, accessed on 25 March 2024). This process included adapter trimming, removal of low-quality bases, exclusion of reads with an N-ratio exceeding 10%, and the discarding of reads shorter than 20 bp. Following quality control, the processed data were aligned to the reference genome of white shrimp (reference genome version: GCF_003789085.1) using HISAT 2.2.1 (accessible at http://ccb.jhu.edu/software/hisat2/index.shtml, accessed on 25 March 2024). This alignment generated mapped reads and allowed for the assessment of mapping quality. Finally, gene and transcript expression levels were quantified using RSEM v1.1.17 (available at http://deweylab.github.io/RSEM/, accessed on 25 March 2024), enabling subsequent differential expression analysis.

### 2.3. Analysis and Functional Annotation of DEGs During Ecdysis

Differential gene expression analysis was performed using DESeq2 1.47.2 (available at http://bioconductor.org/packages/stats/bioc/DESeq2/, accessed on 27 April 2024). A significance threshold of false discovery rate (FDR) < 0.01 and an absolute log2 fold change |log2FC| ≥ 2 were applied to identify key DEGs. An FDR threshold of <0.01 indicates that fewer than 1% of the significant results are expected to be false positives. The |log2FC| ≥ 2 threshold, which corresponds to at least a fourfold difference in gene expression, was implemented to ensure more stringent screening criteria, thereby improving the reliability and accuracy of the DGEs findings. The identified DEGs were subsequently annotated using GO and KEGG pathway analyses facilitated by Diamond v2.1.11 (https://github.com/bbuchfink/diamond, accessed on 28 April 2024). GO annotation categorized the genes into three domains: Biological Process (BP), Cellular Component (CC), and Molecular Function (MF). Meanwhile, KEGG pathway analysis employed ID mapping to associate genes with relevant functional pathways. Enrichment analysis of GO terms was conducted using Goatools (https://github.com/tanghaibao/GOatools, accessed on 7 May 2024), and KEGG pathway enrichment was performed using KOBAS. Statistical significance in these enrichment analyses was assessed using Fisher’s exact test, with *p*-values adjusted for multiple testing corrections. This approach provided valuable insights into the functional roles and biological significance of the DEGs.

### 2.4. Prediction of Neuropeptides Among Differentially Expressed Genes

Based on the typical characteristics of neuropeptides—such as the presence of signal peptides, the absence of transmembrane domains, and adherence to known structural features of arthropod neuropeptides—we employed the following approach to identify key regulatory neuropeptides involved in the molting cycle of the Pacific white shrimp. First, the DEG set was compared against our local database of predicted signal peptides, which was constructed using SignalP 6.0 (https://services.healthtech.dtu.dk/services/SignalP-6.0/, accessed on 10 May 2024). SignalP 6.0 is a highly accurate tool for identifying signal peptides by analyzing the N-terminal sequence of proteins, making it particularly suitable for creating a reliable reference database for secreted proteins, including neuropeptides. To ensure the exclusion of membrane-bound proteins, we predicted non-transmembrane domains using DeepTMHMM 1.0 (https://dtu.biolib.com/app/DeepTMHMM/run, accessed on 10 May 2024), a deep learning-based platform specifically designed for high-accuracy transmembrane domain prediction. Genes predicted to contain signal peptides but lacking transmembrane domains were selected for further analysis. Next, we cross-referenced the insect neuropeptide database nEUROSTRESSPEP and utilized the NeuroPred platform (http://stagbeetle.animal.uiuc.edu/cgi-bin/neuropred.py, accessed on 10 May 2024) to predict neuropeptide cleavage sites, following the methodology outlined by A. E. Christie [19]. NeuroPred predicts neuropeptide cleavage sites by recognizing prohormone processing patterns, which is essential for identifying neuropeptide precursors. This comprehensive workflow enabled the confident identification of DEGs as neuropeptides, providing valuable insights into their potential roles in the molting cycle of the Pacific white shrimp.

### 2.5. RT-qPCR Validation of Key Neuropeptides Regulating Ecdysis

To validate the accuracy of the transcriptomic data, RT-qPCR verification was conducted for the key neuropeptide genes identified in the analysis. Specific primers were designed using Primer Premier 5 software and NCBI Primer-BLAST (https://www.ncbi.nlm.nih.gov/tools/primer-blast/, accessed on 20 May 2024) and subsequently synthesized by Hainan Nanshan Biotechnology Co., Ltd. (Hainan, China). Before RT-qPCR, the primers were tested using high-fidelity PCR, and the resulting amplified products were sequenced by Hainan Nanshan Biotechnology Co., Ltd. to confirm their accuracy. RT-qPCR experiments were performed using SYBR Green I Master Mix from Vazyme Biotech (Matrix, Singapore). To ensure optimal conditions, temperature and concentration gradient assays were carried out to generate standard curves and determine the amplification efficiency for each primer set. The 18S *rRNA* and β-*actin* genes of *P. vannamei* were selected as internal reference genes, with their geometric mean used as the normalization factor [20]. Four replicates were performed for each gene, utilizing parallel samples from the same treatments as in the earlier experiments. The relative expression levels of the genes were calculated using the 2^−ΔΔCT^ method [21], and the log-transformed data were compared with the transcriptomic expression results. After confirming homogeneity of variance, a T-test was used to examine the relative quantitative differences in gene expression before and after molting, with a significance threshold set at *p* < 0.05. All visualizations were created using GraphPad Prism 9.5 (GraphPad Software Inc., San Diego, CA, USA).

## 3. Results

### 3.1. Quality Control of Transcriptome Sequencing Data

The initial experimental design included three samples for sequencing, both before and after molting. However, due to the extended time required for neural isolation, the RNA quality was compromised, resulting in only two samples from each group meeting the sequencing quality standards. A total of 29.47 Gb of clean data was obtained, with individual sample clean data volumes ranging from 6.58 to 8.21 Gb. The percentage of bases with a Q30 score exceeded 94.15%, and the average GC content was approximately 47.45%. Clean reads were successfully mapped to the reference genome, with mapping rates ranging from 87.93% to 88.80% (Table 1). Key metrics, including error rate, genome mapping rate, and clean data yield, all met or exceeded the standard requirements for transcriptome sequencing. These results confirmed that the sequencing data were of high quality and suitable for further analysis.

### 3.2. Annotation and Functional Enrichment of DEGs Before and After Molting

**Figure 1:**
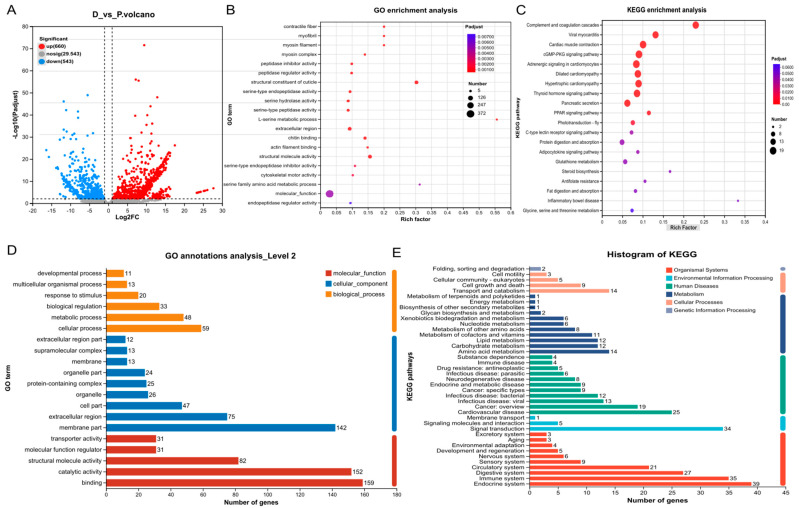
Differentially expressed genes (**A**) before molting (D2 and D3) and after molting (**A**,**B**) and their GO functional enrichment (**B**), KEGG functional enrichment (**C**), GO annotation (**D**), and KEGG functional annotation (**E**).

Transcriptomic differential expression analysis of *P. vannamei* before and after molting is presented in the volcano plot (Figure 1A). Among the 30,746 genes analyzed, 1203 were identified as differentially expressed, comprising 660 upregulated genes and 543 downregulated genes (Figure 1A). GO enrichment analysis highlighted the most significantly enriched pathways for the DEGs, including molecular function (372 DEGs), structural molecule activity (82 DEGs), and structural constituent of the cuticle (81 DEGs) (Figure 1B). KEGG enrichment analysis revealed that the most significantly enriched pathways included the complement and coagulation cascades (18 DEGs), the GMP-PKG signaling pathway (19 DEGs), and several pathways related to nutrient digestion and metabolism (Figure 1C). Of the 1203 DEGs, 1073 were annotated in the GO database, with the most prominent pathways being binding (159 DEGs), catalytic activity (152 DEGs), and membrane part (142 DEGs) (Figure 1D). In the KEGG annotation, 144 DEGs were assigned to pathways, with the most significant being the endocrine system (39 DEGs), immune system (35 DEGs), and signal transduction (34 DEGs) (Figure 1E). Both functional enrichment and annotation analyses revealed that the GO database provided greater utility in this study, making it the preferred resource for further neuropeptide analysis.

### 3.3. Selection and Functional Enrichment of Neuropeptides

**Figure 2:**
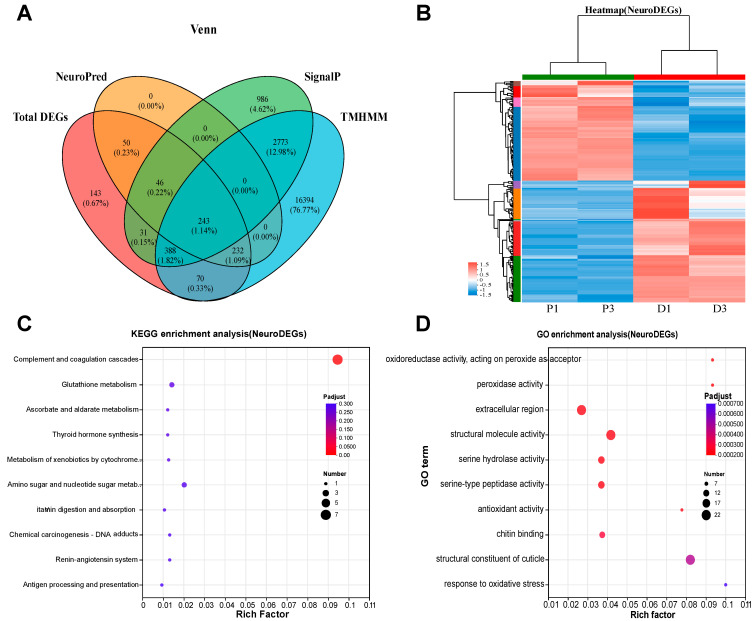
Based on neuropeptide prediction, signal peptide, and transmembrane structure screening of differential neuropeptides involved in molting (**A**) and their expression heat map (**B**), KEGG enrichment (**C**), and GO enrichment analysis (**D**). NeuroPred represents differential neuropeptide genes predicted by NeuroPred; SignalP represents differential genes with signal peptides; TMHMM represents differential genes without transmembrane structures.

To screen neuropeptides related to molting, neuropeptide prediction tools were used, focusing on specific characteristics such as the presence of signal peptides and the absence of transmembrane domains. Among the 30,746 proteins identified in *P. vannamei*, 4467 proteins were predicted to contain signal peptides, while 20,100 genes were predicted to lack transmembrane domains. Neuropeptide predictions were conducted on 1203 DEGs, resulting in the identification of 571 proteins as potential neuropeptides. Using these data, a Venn plot was generated, identifying 243 differentially expressed neuropeptides that possess signal peptides but lack transmembrane domains (Figure 2A). Heatmap analysis of these 243 neuropeptides showed that most genes displayed consistent expression patterns within their respective groups, although a subset of genes exhibited opposing expression patterns within the same group (Figure 2B). Functional enrichment analysis of the 243 neuropeptides was performed using GO and KEGG pathways. GO analysis highlighted several major categories among the top 10 enriched terms, including “extracellular region” (22 DEGs), “structural molecule activity” (22 DEGs), and “structural constituent of cuticle” (22 DEGs) (Figure 2D). In contrast, KEGG pathway enrichment analysis revealed fewer genes in the top 10 pathways, with notable pathways such as “Complement and coagulation cascades” (7 DEGs) (Figure 2C).

### 3.4. Identification of Key Neuropeptides Associated with Molting

**Figure 3:**
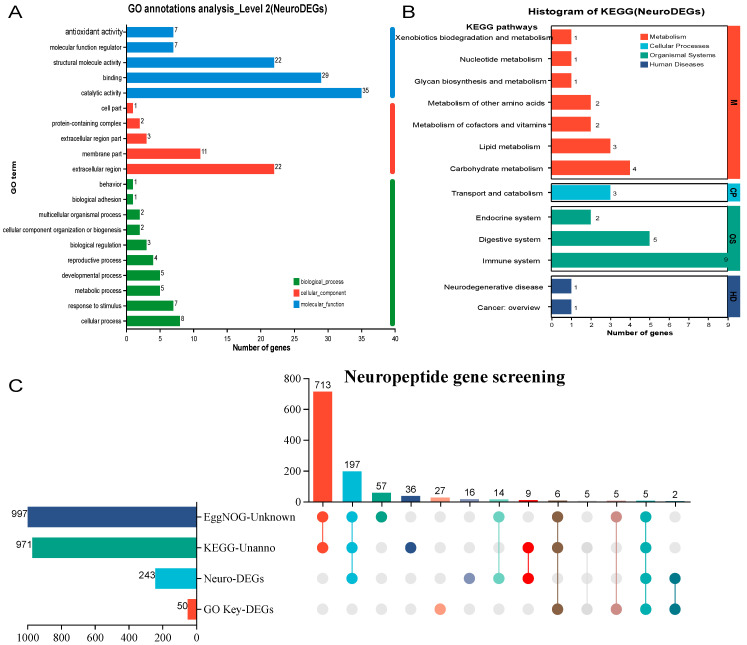
KEGG annotation (**A**), GO annotation (**B**), and upset plot of key neuropeptide gene screening (**C**) for molting-regulated neuropeptides.

Of the 1203 DEGs identified before and after molting, only 232 genes were annotated in the KEGG database, leaving 971 genes unannotated, indicating that over 80% of the DEGs lacked annotation (Figure 3A,C). Similarly, among the 1203 DEGs screened from the transcriptome data, 997 genes were either unannotated in the EggNOG database or were annotated as “Function unknown” (Figure 3C). In contrast, the GO annotation provided a broader scope of functional insights in this study, with the top 10 pathways alone encompassing 50 neuropeptides (Figure 3B,C). To further explore novel neuropeptides related to molting that have not been previously reported, genes unannotated by EggNOG and KEGG, along with those annotated to key pathways by GO, were collectively analyzed to construct an upset plot. This approach helped identify key neuropeptides (Figure 3C). Based on this analysis, five key genes were identified, corresponding to the gene numbers LOC113823331, LOC113815764, LOC113819918, LOC113817637, and LOC113818077. These genes were named *PvCHH*, *PvMIH*, *PvEH I*, *PvCDA I*, and *PvCDA II*, respectively (Table 2). When compared to the NCBI database, the aligned sequences were identified as follows: *P*. *vannamei* crustacean hyperglycemic hormone 6-like (*PvCHH*), *P*. *vannamei* molt-inhibiting hormone-like transcript variant X1 (*PvMIH*), *P*. *vannamei* eclosion hormone-like transcript variant X2 (*PvEH I*), *Penaeus chinensis* chitin deacetylase 1-like (*PvCDA* I), and *Penaeus monodon* chitin deacetylase 1-like (*PvCDA* II) (Table 2). The *PvCHH*, *PvMIH*, and *PvEH I* genes showed 100% sequence identity with the *P. vannamei* gene database. However, *PvCDA I* and *PvCDA II* were not found in the *P. vannamei* gene database. Instead, their homologous sequences were identified in *P. chinensis* and *P. monodon*, with sequence identities of 88% and 94%, respectively (Table 2).

### 3.5. RT-qPCR Primer Design, Amplification Efficiency, and Validation

**Figure 4:**
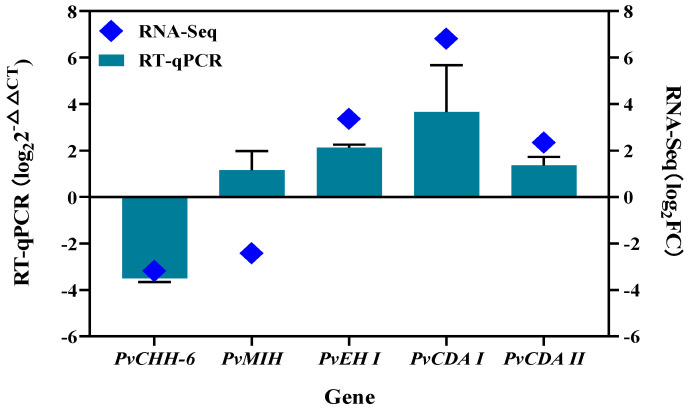
RT-qPCR validation of transcriptome screening for key neuropeptide genes.

Primers were designed based on the obtained sequences and are listed in Table 3. Before conducting further analyses, all primers were validated through PCR amplification and sequencing to confirm the accuracy of the target sequences. The optimal amplification temperatures and efficiencies for the RT-qPCR primers of *PvCHH*, *PvMIH*, *PvEH I*, *PvCDA I*, and *PvCDA II*, as well as the internal reference genes *18S rRNA* and *β-actin*, were determined. The optimal annealing temperatures were 63.3 °C, 63.3 °C, 63.3 °C, 63.3 °C, 64.7 °C, 60.6 °C, and 65.9 °C, respectively, with amplification efficiencies of 91.5%, 93.5%, 94.5%, 97.5%, 97.5%, 97.0%, and 92.5% (Table 3). These results demonstrate that the primers used in this study specifically amplified the target genes and were suitable for quantification using the 2^−ΔΔCT^ method. RT-qPCR was performed to validate the differential expression of five key neuropeptide genes identified from transcriptome analysis. The results are shown in Figure 4. The expression patterns of *PvCHH*, *PvEH I*, *PvCDA I*, and *PvCDA II* were consistent with the transcriptome data. However, *PvMIH* showed an upregulated expression in RT-qPCR compared to a down-regulated expression in the transcriptome during the pre-molt stage relative to the post-molt stage (Figure 4).

## 4. Discussion

This study aimed to investigate the key neuroregulatory mechanisms underlying the molting process of *P*. *vannamei*. Through comprehensive transcriptomic analysis, we successfully identified critical pathways related to molting. By leveraging features such as signal peptides, the absence of transmembrane domains, and neuropeptide prediction, we identified 243 DEGs as neuropeptides. Furthermore, we identified five novel gene sequences—*PvCHH*-6, *PvMIH*, *PvEH I*, *PvCDA I*, and *PvCDA II*—that exhibited significant differential expression during the molting process. These sequences were not previously annotated in the KEGG or NOG databases, suggesting their potential as key regulators in the molting of *P. vannamei*. The identification of these neuropeptide genes enhances our understanding of neuropeptide-mediated regulation in shrimp molting and provides a valuable foundation for future functional studies.

GO enrichment analysis of differentially expressed genes revealed that the most significantly enriched molecular functions included binding, catalytic activity, and structural molecule activity, indicative of the regulation of newly formed shells or their molecular components, metabolic activity, and protein functions associated with signal transduction during the molting process. Binding activity may involve ligand-receptor interactions that regulate cellular signaling pathways [22], while catalytic activity is likely implicated in the regulation of metabolic pathways and the transformation of substrates. During molting, the secretion and hardening of the new exoskeleton require membrane reorganization and metabolic regulation, and genes enriched in the extracellular region may contribute to shell formation [23]. The synthesis and degradation of proteins, as well as the regulation of the cell cycle, demand significant energy and intracellular activity during molting. This may account for the observed enrichment of genes involved in cellular and metabolic processes [24,25]. Furthermore, the enrichment of neuropeptide-related genes in functions such as cuticle structural constituents, structural molecule activity, and the extracellular region underscores the pivotal role of neuropeptides in regulating the formation and hardening of the new exoskeleton. These genes also appear to be critical for the repair and protection of the shrimp’s body during molting [11,26].

Neuropeptides play a pivotal role in various metabolic processes; however, research on their specific functions remains limited. Transcriptomic analysis, particularly through high-throughput sequencing, has emerged as an effective tool for uncovering gene regulatory mechanisms. This approach has been successfully applied to various aspects of *P. vannamei* biology, including reproduction, physiology, and nutritional requirements, yielding significant insights [16,27,28]. However, the scope of current transcriptomic techniques for revealing differential gene expression remains somewhat constrained. Advanced predictive methods, such as those leveraging deep learning models, have demonstrated considerable promise in fields like proteomics, genome annotation, and the identification of potential drug targets. A critical aspect of interpreting protein functions is understanding their secretion or membrane anchoring mechanisms [29]. In this study, we integrated specific features—such as signal peptides, the absence of transmembrane domains, and neuropeptide prediction—into our transcriptomic analysis [19,30,31,32], identifying 243 neuropeptide-related genes that may play vital roles in the molting process of *P. vannamei*. These findings will facilitate further exploration of neuropeptide regulation in shrimp molting.

The X-organ-sinus gland (XO-SG) complex, a crucial neuroendocrine organ in crustaceans, serves a functional role analogous to the hypothalamus-pituitary system in vertebrates. This complex secretes various neuropeptides to regulate physiological activities [19], with CHH being one of the most abundantly secreted neuropeptides [13]. The CHH family plays significant roles in numerous physiological functions, including glucose regulation, osmoregulation, molting, growth, reproduction, and immunity [33,34,35,36]. A defining feature of CHH family neuropeptides is the presence of six highly conserved cysteine residues that form three disulfide bonds [37,38]. Among these, MIH is a notable member of the CHH family. MIH regulates molting by acting on the Y-organ to modulate the production of molting hormones, thereby influencing the timing of molting initiation or delay [36,39]. In *P. vannamei*, *MIH* has been identified, with two genes reported [12], and its regulatory role in the molting process of this species has been confirmed [12]. In this study, two of the five key genes identified (*PvCHH-6* and *PvMIH*) belong to the CHH family, reflecting the reliability of our research methods.

Moreover, we identified the gene encoding eclosion hormone (EH), which is responsive to the molting hormone (20E) during the later stages of molting. In *Exopalaemon carinicauda*, the administration of exogenous 20E was found to upregulate *EH* expression [40]. Extensive research has demonstrated that EH likely plays a direct role in regulating molting through a series of cell signaling pathways [40,41,42,43]. In arthropods, EH is essential for successful molting, and its deficiency can lead to significant disruptions in the molting process. For instance, *Drosophila* mutants and *EH* knockouts in *Tribolium castaneum* exhibited high rates of pre-molt mortality [44,45]. Similarly, in crustaceans such as *Scylla paramamosain* [46] and *Exopalaemon carinicauda* [40], disruptions in EH function resulted in delayed molting and increased mortality. These findings underscore the highly conserved role of EH in the molting processes across arthropods.

Chitin plays an essential role in the development and molting processes of arthropods. The deacetylation of chitin enhances the solubility of chitin fibers, reducing their density and influencing their structural integrity within the exoskeleton [47]. Furthermore, chitin deacetylases may contribute to immune responses or alleviate the inhibitory effects of chito-oligo saccharides on chitinase activity, a function that is particularly vital during molting [48]. In *Drosophila melanogaster*, chitin deacetylase proteins CDA1 and CDA2 are involved in tracheal extracellular matrix formation and limit tube elongation [49,50]. Research on *Diaphorina citri* has further demonstrated that chitin deacetylase (CDA) is involved in the regulation of chitin and fatty acid metabolism, with its expression being induced by 20-hydroxyecdysone (20E) [51]. Moreover, RNA interference (RNAi)-mediated suppression of CDA has been shown to severely disrupt the molting process in both *Diaphorina citri* and *Holotrichia parallela*, leading to increased mortality and higher rates of deformities [52].

This study utilized an innovative approach to identify key neuropeptides involved in the molting regulation of *P. vannamei*, providing foundational data for the future application of neuropeptide regulation in shrimp aquaculture. However, due to the rapid fluctuations in neuropeptide secretion during molting, some samples were excluded from subsequent analyses, resulting in a final dataset comprising only two biological replicates. While this limitation may impact the robustness of the findings, we implemented stringent selection and validation criteria, including FDR < 0.01 and |log2FC| ≥ 2, to ensure the reliability of the results. This study offers valuable insights and establishes a methodological framework for future research on shrimp molting. For instance, our team recently demonstrated the critical role of EH in the molting process of *P. vannamei* using RNAi technology [18]. Building on these findings, further investigations—such as the synthesis and application of active proteins, as well as the use of CRISPR or RNAi techniques—will enhance our understanding of the functions of key neuropeptides and promote their application in shrimp aquaculture management.

## 5. Conclusions

This study elucidates the critical neuroregulatory mechanisms underlying the molting process in *P*. *vannamei* through comprehensive transcriptomic analysis. By identifying 1203 DEGs, we uncovered significant pathways associated with molting, with a particular emphasis on the roles of neuropeptides. GO and KEGG enrichment analyses highlighted key pathways, including the complement and coagulation cascades, the GMP-PKG signaling pathway, and functions related to the structural constituents of the cuticle. Notably, the discovery of five novel neuropeptide genes—*PvCHH*, *PvMIH*, *PvEH I*, *PvCDA I*, and *PvCDA II*—underscores their potential regulatory roles during molting. These key neuropeptides are expected to provide critical insights into the regulatory mechanisms of molting and serve as valuable targets for the development of synthetic analogs. Such synthetic products hold significant promise for improving molting uniformity and efficiency in shrimp aquaculture, thereby supporting the industry’s sustainable growth and productivity.

## Figures and Tables

**Table 1:** Sample data quality summary.

Sample	Raw Reads	Clean Reads	Total Mapped	Error Rate (%)	Q20 (%)	Q30 (%)	GC Content (%)
D1	44,354,688	43,993,916	39,066,617 (88.8%)	0.0245	98.27	94.58	46.08
D2	45,314,592	44,840,126	39,694,826 (88.53%)	0.0246	98.23	94.48	47.39
P1	55,634,454	55,031,736	48,487,337 (88.11%)	0.025	98.1	94.15	48.28
P2	53,708,600	53,274,568	46,846,281 (87.93%)	0.0243	98.35	94.77	48.06

Notes: Samples D1 and D2 are samples collected during the pre-molt stage, while P1 and P2 are samples collected during the post-molt stage; Raw reads: raw sequencing data entries; Clean reads: quality-controlled sequencing data entries; Total mapped: the number of clean reads mapped to the genome; Error rate (%): average error rate of bases in quality-controlled data; Q20 (%), Q30 (%): quality assessment metrics for sequencing data; GC content (%): percentage of G and C bases in quality-controlled data.

**Table 2:** Information of neuropeptide genes.

Gene ID/mRNA	Gene Description	Identity (%)	Name
LOC113823331 XM_027375947.2	*Penaeus vannamei* crustacean hyperglycemic hormone 6-like	100	*PvCHH*
LOC113815764 XM_027367780.2	*Penaeus vannamei* molt-inhibiting hormone-like, transcript variant X1	100	*PvMIH*
LOC113819918 XM_027372151.2	*Penaeus vannamei* eclosion hormone-like, transcript variant X2	100	*PvEH I*
LOC113817637 XM_047637281.1	*Penaeus chinensis* chitin deacetylase 1-like	88	*PvCDA I*
LOC113818077 XM_037923701.1	*Penaeus monodon* chitin deacetylase 1-like	94	*PvCDA II*

**Table 3:** Primers used to verify the accuracy of transcriptome data.

NCBI ID	Primer	Sequence (5′ to 3′)	TM (°C)	PCR Efficiency (%)
LOC113823331	*PvCHH-F*	AAGATCGCCTTCGTCTCTGC	63.3	91.5
	*PvCHH-R*	CGTCGAAGACCTGCCTCTTT		
LOC113815764	*PvMIH-F*	TTGAGAAGCTGCTGTCGTCC	63.3	93.5
	*PvMIH-R*	GCGTAGCAGTTACTCTTGCAC		
LOC113819918	*PvEH I-F*	GCTGATGTACCACGACCACT	63.3	94.5
	*PvEH I-R*	AATGAGGTCCTGTGGGTTCG		
LOC113817637	*PvCDA I-F*	CAACTCGTTCGAACCCTGGA	63.3	97.5
	*PvCDA I-R*	ACTCGTTCTTGAGCCAAGGG		
LOC113818077	*PvCDA II-F*	TGGGGCTTCCTCTACGACT	64.7	97.5
	*PvCDA II-R*	GACACTTGTGGGGCATACG		
AF186250	*Pv18S rRNA-F*	TATACGCTAGTGGAGCTGGAA	60.6	97.0
	*Pv18S rRNA-R*	GGGGAGGTAGTGACGAAAAAT		
AF300705	*Pvβ-actin-F*	CGAGAAATCGTTCGTGAC	65.9	92.5
	*Pvβ-actin-R*	GATGGAGTTGTAGGTGGTCT		

## Data Availability

Data will be made available on request.
